# Longitudinal Associations of Physical Touch with Loneliness among Older Adults

**DOI:** 10.1093/geroni/igab046.3711

**Published:** 2021-12-17

**Authors:** Taylor Brown, Reese Giddens, Stephanie Wilson

**Affiliations:** 1 Southern Methodist University, Dallas, Texas, United States; 2 Southern Methodist University, Southern Methodist University, Texas, United States; 3 Southern Methodist University, DALLAS, Texas, United States

## Abstract

Older adults in the US face heightened risks for social disconnection, and the COVID-19 pandemic has further exacerbated this crisis. Physical touch is a key dimension of social connection that uniquely predicts physical and mental health benefits. However, most studies have been limited by cross-sectional designs, and no prior work has examined the long-term effects of physical touch on loneliness. To investigate the prospective association between physical touch and loneliness among older adults, this study utilized data from 1626 older adults (Mean age = 68, range = 57-85) who participated in Waves 1 and 2 of the National Social Life, Health, and Aging Project (NSHAP). Participants reported on their loneliness and physical contact with family and friends, as well as with pets, at both waves. Results revealed that more frequent physical contact with family and friends predicted larger decreases in loneliness over the subsequent five years (p<.0001), controlling for age, race, gender, health conditions, marital status, frequency of social interaction, and baseline levels of loneliness. Physical contact with pets had no unique effect (p=.136). To further assess directionality, models tested whether lonelier people experienced decreased touch over time, and the effects were null (p>.250). Taken together, this longitudinal study is the first to identify the unique contribution of human physical touch to prospective changes in loneliness, beyond the well-established effects of covariates, including social interaction frequency. Touch represents a compelling mechanism by which social isolation may lead to loneliness, which in turn raises risks for poor health and premature mortality.

